# Técnicas para el tratamiento del queratoquiste, revisión de la literatura y presentación de un caso

**DOI:** 10.21142/2523-2754-1102-2023-159

**Published:** 2023-06-29

**Authors:** Jorge Andrés Ochoa Moreira, Santiago José Reinoso Quezada, Magdalena Molina-Barahona

**Affiliations:** 1 Universidad Católica de Cuenca. Cuenca, Ecuador. jorg8c@hotmail.com, sreinoso@ucacue.edu.ec, rocio.molina@ucacue.edu.ec Universidad Católica de Cuenca Universidad Católica de Cuenca Cuenca Ecuador jorg8c@hotmail.com sreinoso@ucacue.edu.ec rocio.molina@ucacue.edu.ec

**Keywords:** queratoquiste odontogénico, tumor odongénico queratoquístico, tratamiento, recurrencia, técnica, odontogenic keratocyst, odontogenic keratocystic tumor, treatment, recurrence, technique

## Abstract

El tumor queratoquístico odontogénico (TQO), también conocido actualmente como queratoquiste odontogénico (QQO), es una patología benigna derivada de los restos de la lámina dental, caracterizada por poseer cantidades variables de paraqueratina descamada y se presenta de forma solitaria o con quistes satélite. La aparición de estos quistes suele estar relacionada con la posible recurrencia del QQO y, según la literatura, esta recidiva puede variar entre el 0% y el 50%. En cuanto a la etapa de tratamiento del QQO, se puede mencionar que, en la actualidad, existe un criterio histológico y clínico bien definido, lo que facilita su reconocimiento y, por ende, su tratamiento. Existen varias modalidades de tratamiento, las cuales pueden ser clasificadas en tratamientos no conservadores o radicales, y conservadores acompañados de métodos adyuvantes. Entre los tratamientos no conservadores o radicales encontramos la resección en bloque, que es la forma más agresiva de tratar un queratoquiste; sin embargo, se ha demostrado que es la forma más eficaz para evitar la recidiva. Por su parte, entre los tratamientos conservadores se describe a la marsupialización, descompresión y enucleación, con o sin terapia adyuvante. Es importante reconocer los distintos tipos de tratamientos para el QQO, ya que este estará condicionado por múltiples factores como la localización en relación con sus estructuras óseas cercanas y el tamaño de la lesión teniendo en cuenta la posible afección de estructuras dentales. El objetivo es buscar el tratamiento de menor riesgo posible, que evite recurrencia y, finalmente, acabe con esta patología.

## Abstract (en)

The odontogenic keratocystic tumor (OKT) or also currently known as odontogenic keratocyst (OK) is a benign pathology derived from the remains of the dental lamina characteristic for possessing variable amounts of desquamated keratin. It usually rises as solitary or with the presence of satellite cysts, the appearance of these satellite cysts is frequently related to the possible recurrence of OK, according to the literature, this recurrence can vary between 0 - 50%. As for the treatment stage of (OKT), it can be mentioned that at present there is a well-defined histological and clinical criterion, which facilitates its recognition and therefore its treatment. According to the literature, there are several treatment procedures that can be classified into non-conservative or radical treatments and conservative treatments accompanied by adjuvant methods. Within the non-conservative or radical treatments, we find en bloc resection, which is the most aggressive way to treat a keratocyst; however, it is the most effective way to avoid recurrence. Within the conservative treatments, marsupialization, decompression, and enucleation with or without adjuvant therapy are described. It is paramount to know how to recognize the different types of treatment for (OKT) since this will be conditioned by multiple factors, such as the location of nearby bone structures and the size of the lesion considering the possible involvement of dental structures. The objective is to seek the lowest-risk treatment possible, which avoids recurrence and finally puts an end to this pathology.

## INTRODUCCIÓN

El tumor queratoquístico odontogénico (TQO), también conocido actualmente como queratoquiste odontogénico (QQO) -debido a la última clasificación de “lesiones de quistes odontogénicos y no odontogénicos del desarrollo” de la OMS (2022) ^(1, 2)^-, es una lesión derivada de los restos de la lámina dental (restos de Serres) de origen benigno, con un comportamiento similar a otros quistes odontogénicos; sin embargo, se caracteriza por ser de carácter más agresivo y recurrente ^2^^-^^4^. Según la literatura, el QQO tiene una incidencia de un 3-11% de los quistes maxilares y su afección es predominante en hombres, con una proporción 2:1 en comparación con las mujeres. Esta lesión se presenta en un rango de edad entre la segunda y tercera década de vida, con un segundo pico de incidencia entre la quinta y sexta década de vida. Aunque cualquier hueso, maxilar o mandíbula puede sufrir esta lesión, la mayoría de veces la mandíbula tiene una predominancia 2:1 y hasta en un 75% esta lesión se encuentra en la región posterior de la mandíbula ^1^^,^^5^^-^^8^.

Clínicamente, el QQO se presenta como una lesión solitaria excepto cuando se asocia al síndrome de Gorlin-Goltz o síndrome de nevo basocelular, caracterizado por presentar varias lesiones neoplásicas ^1^^,^^3^^,^^4^^,^^8^. Es importante recalcar que, en su etapa clínica inicial, el QQO no presenta sintomatología; generalmente es descubierto debido a exámenes complementarios solicitados por el odontólogo. No obstante, en etapas avanzadas, puede manifestarse mediante expansión bucal o lingual, dolor, hinchazón o secreción, y en raras ocasiones parestesia del labio inferior ^3^.

Radiográficamente su manifestación es de predominancia radiolúcida unilocular o multilocular, similar a un “panal de abejas”. En un 25-40% de los casos se suelen manifestar dientes no erupcionados ^1^^,^^7^^,^^8^.

Histopatológicamente, el QQO se caracteriza por poseer las siguientes características: 1) recubrimiento delgado y uniforme de epitelio escamoso paraqueratinizado; por lo general, de 6 a 10 células de espesor; 2) una capa de paraqueratina ondulada en su superficie luminal; 3) una capa en empalizada de células basales cuboides o prismáticas; 4) ausencia de papilas; y 5) la luz del quiste es característica por poseer cantidades variables de paraqueratina descamada ^3^^,^^4^^,^^8^. Otros rasgos para tomar en cuenta dentro de la histopatología son la presencia de residuos de la lámina dental y la formación de microquistes o quistes satélites, la cual suele estar relacionada con la posible recurrencia del QQO. Según la literatura, esta puede variar entre el 0% y el 50% ^1^^,^^3^^,^^4^.

**Figura 1:**
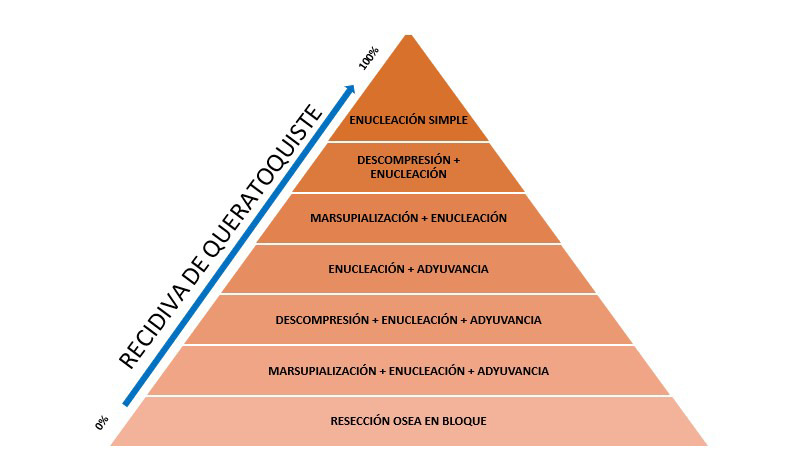
Pirámide que indica tratamientos del queratoquiste odontogénico y nivel de recidiva según el procedimiento

En cuanto a la etapa de tratamiento del QQO, se puede mencionar que, en la actualidad, existe un criterio histológico y clínico bien definido, lo que facilita su reconocimiento y, por ende, su tratamiento. Existen varias modalidades de tratamiento, las cuales pueden ser clasificadas en tratamientos no conservadores o radicales, y tratamientos conservadores acompañados de métodos adyuvantes ^4^^,^^9^. Entre los tratamientos no conservadores o radicales encontramos la resección en bloque, que es la forma más agresiva de tratar un queratoquiste; sin embargo, se ha demostrado que es la más eficaz para evitar la recidiva en la lesión. Por su parte, entre los tratamientos conservadores se describe a la marsupialización, descompresión y enucleación, con o sin terapia adyuvante (crioterapia, osteotomía periférica o aplicación de la solución de Carnoy) ^1^^,^^3^^,^^6^^,^^9^ (Fig. 1). 

Es importante tener presente que el tipo de tratamiento para el QQO está condicionado por múltiples factores, como la localización en relación con sus estructuras óseas cercanas, el tamaño de la lesión, la posible afección de estructuras dentales, entre otros. El objetivo es buscar el tratamiento de menor riesgo posible, que evite recurrencia y, finalmente, acabe con la lesión ^6^^,^^9^.

## TIPOS DE TRATAMIENTOS

### 1. Terapias adyuvantes

Los tratamientos o terapias adyuvantes se pueden utilizar como un complemento a los tratamientos conservadores y no conservadores del QO, con el propósito de eliminar los posibles restos epiteliales de la pared quística que puedan quedar en el hueso adyacente y alcancen a estimular la reincidencia. Entre estas terapias se pueden describir las siguientes:

#### 1.1. Crioterapia

También conocida como criocirugía, es un método muy poco utilizado, complementario a la enucleación o resección del QQO, en el cual soluciones como el nitrógeno líquido a 20 °C inducen a la necrosis celular en el hueso, manteniendo fragmentos óseos inorgánicos que, a futuro, ayudan a la regeneración ósea en la zona a tratar. Es importante recalcar su manejo, ya que, en tejido sano, puede mostrar desvitalización ^10^.

#### 1.2. Osteotomía periférica

Posee características complementarias a la enucleación y tiene como objetivo eliminar tejido óseo de entre 1,5 y 2 mm del margen quirúrgico, para evitar cualquier residuo de tejido queratoquístico a nivel óseo. Este tratamiento y la solución de Carnoy son los más utilizados como prevención de la recidiva ^1^^,^^4^.

#### 1.3. Solución de Carnoy

Tiene como objetivo impedir la recidiva de la lesión mediante su penetración a través del margen óseo de entre 1 y 1,5 mm. Esta solución no debe exceder los cinco minutos para evitar su función como inductor de neurotoxicidad local, lo cual depende mucho del tiempo de exposición. Está constituida por alcohol (6 ml), cloroformo (3 ml), ácido acético (1 ml) y clorhídrico férrico (0,1 mg) ^1^^,^^4^^,^^6^.

La colocación de la solución se debe realizar posterior a la enucleación del QQO, el curetaje y la limpieza del nicho quirúrgico, y no debe tener contacto con tejidos circundantes ^4^^,^^6^.

### 2. Tratamientos conservadores.

#### 2.1. Enucleación

**Figura 2:**
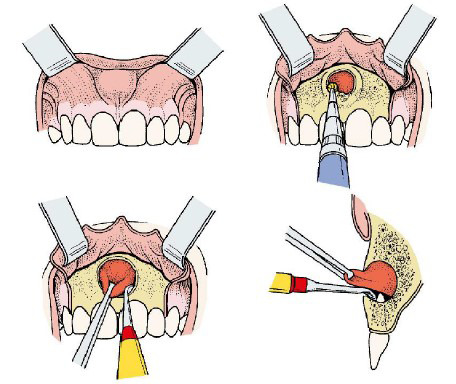
Ilustración de la enucleación de un quiste en la zona maxilar ^25^^).^

Consiste en eliminar la cápsula quística de la lesión sin comprometer las estructuras anatómicas adyacentes ni la continuidad del maxilar o la mandíbula ^1^^,^^4^ (Fig. 2). 

#### 2.2. Descompresión

Busca la liberación del componente quístico mediante la inserción de una cánula de polietileno; este tratamiento propone producir cambios en el epitelio quístico a fin de alterar la capa de epitelio queratinizado a uno no queratinizado ^1^^,^^4^^,^^10^.

#### 2.3. Marsupialización

Esta técnica enfoca la externalización del quiste mediante una ventana quirúrgica en la mucosa bucal y la pared quística, donde los márgenes del quiste-mucosa son suturados para crear una ventana abierta que se comunica con la cavidad oral ^1^^,^^4^^,^^9^.

Estos procedimientos (descompresión y marsupialización) buscan aliviar la presión del líquido quístico, con el propósito de reducir el espacio del quiste y favorecer la aposición ósea en las paredes del quiste. Si bien son muy similares, su diferencia reside en el uso de un dispositivo cilíndrico o de un drenaje quirúrgico rígido para evitar el cierre mucoso (descompresión) ^1^^,^^4^^,^^9^.

### 3. Tratamiento no conservador o radical

#### 3.1. Resección en bloque

**Figura 3:**
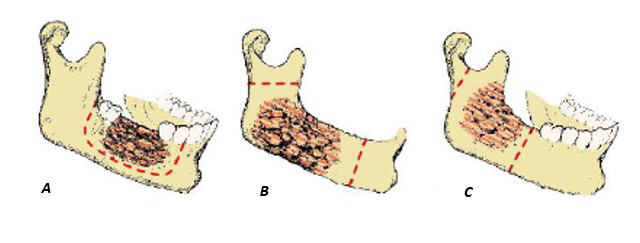
Tipos de resección mandibular ante la presencia de un queratoquiste odontogénico. A) Resección marginal o segmentaria, mantiene mínima estructura ósea. B y C) Resección mandibular en bloque, pierde continuidad del hueso ^25^

La resección segmentaria o en bloque se define como el corte del hueso manteniendo mínimamente su estructura o perdiendo la continuidad total del mismo, con el objetivo de eliminar el quiste y la proliferación de sus células, y así impedir una futura recidiva ^1^^,^^4^^,^^10^ (Fig. 3).

## MATERIALES Y MÉTODOS

En la presente revisión, se evaluaron los artículos de acuerdo con las pautas PRISMA, incluyendo los siguientes puntos:

1) Pregunta de investigación: ¿Existe evidencia científica que detalle el éxito de los tratamientos quirúrgicos actuales aplicados ante la presencia de un queratoquiste odontogénico?

2) Población de estudio: la población de provecho en esta revisión corresponde a pacientes de cualquier edad mencionados en revisiones bibliográficas, sin ningún tipo de síndrome relacionado con el queratoquiste odontogénico y cuyo diagnóstico sea únicamente este. 

3) Tipo de tratamiento y comparación: el tipo de tratamiento a evaluar en la revisión fueron estudios que comparen una o más intervenciones quirúrgicas en pacientes con diagnóstico de QQO, incluidas la enucleación simple, la enucleación con curetaje, la enucleación con terapia adyuvante (crioterapia, osteotomía periférica y Solución de Carnoy), la marsupialización o la descompresión y la resección en bloque.

4) Selección de literatura: los artículos de revisión seleccionados incluyeron estudios retrospectivos, estudios prospectivos y ensayos clínicos controlados que hayan evaluado y comparado varias modalidades de tratamiento quirúrgico con respecto al porcentaje de recurrencia del queratoquiste odontogénico, con un periodo de seguimiento no menor a los 12 meses. 

5) Criterios de inclusión: artículos que presenten diagnóstico confirmado de sus pacientes únicamente con queratoquiste odontogénico, artículos que manifiesten los tratamientos efectuados y el porcentaje de su recidiva, artículos que presenten información publicada en revistas científicas entre enero de 2012 y enero de 2022.

6) Criterios de exclusión: artículos que presenten estudios *in vitro* o en animales, cartas al editor, artículos con muestreo de pacientes inferior a 10, artículos que se relacionen con el síndrome de Gorlin-Gotz (síndrome névico basocelular), artículos que demuestren un periodo de seguimiento menor a 12 meses y artículos que no estén escritos en idioma inglés o español. 

7) Estrategia de búsqueda: se realizó una búsqueda bibliográfica en diferentes fuentes de información científica, como PubMed, Medigraphic, SciELO, Google Académico y Elsevier, con un tiempo de publicación entre enero de 2012 y enero de 2022, y con una combinación de términos de texto libre. Se empleó la siguiente estrategia de búsqueda según los términos MeSH/DeCS: “Odontogenic keratocyst” AND “Treatment” y “Queratoquiste” AND “Tratamiento quirúrgico”. Para evitar el sesgo de selección de artículos, un segundo revisor ayudó a la clasificación de los artículos.

8) Metodología de revisión: Después de realizar una búsqueda inicial exhaustiva de literatura que abarcó textos completos, esta fue llevada a cabo por un solo revisor independiente (Ochoa) y los estudios que cumplieron los criterios de inclusión fueron seleccionados. En caso de existir duda respecto de algún artículo, fue consultado un segundo revisor (Reinoso), para evitar sesgos. El acuerdo completo de los revisores fue esencial para completar esta revisión. 

9) Resultados: se evaluaron todos los tratamientos manifestados según la literatura y su porcentaje de recidiva. 

## RESULTADOS

**Figura 4:**
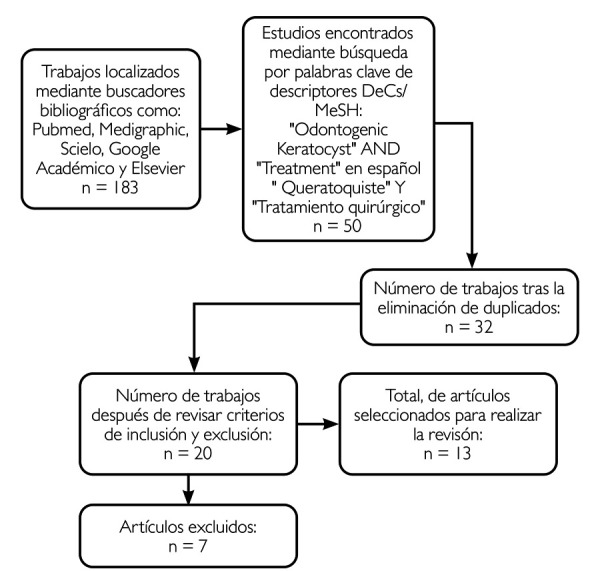
Procedimiento para la selección de artículos

Esta revisión de literatura proporcionó 183 artículos después de aplicar una búsqueda inicial. De ellos, 133 fueron descartados por su título y resumen, idioma e inaccesibilidad al texto; 32 fueron eliminados por ser duplicados y quedaron 20, de los cuales 7 fueron excluidos por no cumplir con criterios de inclusión, lo que dio como resultado final 13 artículos (Fig. 4).

Según la revisión, un total de 2159 pacientes fueron intervenidos, de los cuales 1343 fueron hombres y 816, mujeres. En cuanto a la localización de la lesión, el 78,9% se presentó en la zona mandibular, frente al 21% en la zona maxilar. Cabe recalcar que la mayoría de los estudios fueron retrospectivos (12) y solo uno, de cohorte (Tabla 1).

**Tabla 1:** Datos generales de autor, diseño de estudio, muestreo, sexo y localización de la lesión

Datos generales, pacientes y lesión					
Autor y año	Diseño del estudio	Muestra	Sexo	Localización		
				Mandíbula	Maxilar
Ribeiro Junior *et al*., 2012	Estudio retrospectivo	14 pacientes	7 H (50%) 7M (50%)	72,7% (16)	27,3% (6)
Zhaoo *et al*., 2012	Estudio retrospectivo	19 pacientes	12 H (63%) 7 M (37%)	78,9% (15)	21,1% (4)
Tintichi *et al*., 2012	Estudio retrospectivo	106 pacientes y 145 lesiones	90 H (62,2%) 55 M (37,8%)	75,2% (109)	24,8% (36)
Selvi *et al*.,2012	Estudio retrospectivo	22 pacientes	15 H (68,2%) 7 M (31,8%)	77,3% (109)	22,7% (36)
MacDonald *et al*., 2013	Estudio retrospectivo	29 pacientes	16 H (55%) 13 M (45%)	69% (20)	31% (9)
Levorová *et al*., 2015	Estudio retrospectivo	22 pacientes	13 H (59,1%) 9 M (40,9%)	100% (22)	0% (0)
Gupta *et al*., 2016	Estudio retrospectivo	30 pacientes	23 H (76,7%) 7 M (23,3%)	73,3% (22)	26,7% (8)
Ledderhof *et al*.,2016	Estudio de cohorte	32 pacientes	19 H (59%) 13 M (41%)	84,4% (27)	15,5% (5)
Leung YY *et al*., 2016	Estudio retrospectivo	105 pacientes	54 H (51,4%) 51 M (48,6%)	79% (83)	21% (22)
Dias G *et al*., 2016	Estudio retrospectivo	119 pacientes	73 H (61,3%) 46 M (38,7%)	76,5% (91)	23,5% (29)
Santos de Castro *et al*., 2017	Estudio retrospectivo	1321 pacientes	842 H (63,7%) 479 M (36,3%)	74,5% (1371)	25,5% (529)
Nyimi Bushabu F *et al*., 2019	Estudio retrospectivo	35 pacientes	27 H (77,1%) 8 M (22,9%)	100% (35)	0 % (0)
Jung *et al*., 2021	Estudio retrospectivo	266 pacientes y 274 lesiones	152 H (57%) 114 M (43%)	66% (181)	34% (93)

En los estudios revisados se aplicaron varios tratamientos quirúrgicos, de los cuales la enucleación simple fue el más utilizado (1538 lesiones), seguido por la enucleación más la aplicación de solución de Carnoy (265 lesiones), la descompresión más enucleación (170 lesiones), la marsupialización (122 lesiones), la resección en bloque (54 lesiones), la marsupialización más enucleación (45 lesiones), la enucleación más osteotomía periférica y solución de Carnoy (43 lesiones), la enucleación más curetaje (31 lesiones), la descompresión (27 lesiones), la enucleación más curetaje y solución de Carnoy (11 lesiones) y la enucleación más crioterapia (3 lesiones). En cuanto a la recurrencia según el tipo de tratamiento, la enucleación simple representó el tratamiento con mayor recurrencia, con el 32.9% de todas las lesiones tratadas, comparado con la resección en bloque, cuyo porcentaje fue del 0% de recurrencia.

En la combinación de tratamientos quirúrgicos con terapia adyuvante, estos demostraron tener menor recidiva que cuando se aplica un único tratamiento. Esto indica que la enucleación más la aplicación de solución de Carnoy presentó una recurrencia promedio del 19,3% (Tabla 2).

**Tabla 2:** Datos de autor, técnica de tratamiento, seguimiento y porcentaje de recidiva

Autor y año	Técnica	Tiempo de seguimiento (meses)	Recurrencia		
			# de pacientes	Porcentaje
Ribeiro Junior *et al*., 2012	Enucleación + Solución de Carnoy (22) (100%)	42.9	1	4.55%
Zhaoo *et al*., 2012	Enucleación (133) (51,8%) Enucleación + Solución de Carnoy (124) (48,2%)	12 --180	12 7	9% 5,6%
Tintichi *et al*., 2012	Marsupialización (5) (7,7%) Enucleación (50) (76,9%) Enucleación + Solución de Carnoy (9) (13,8%) Resección (1) (1,5%)	13 23,5 12,6 30	3 15 1 0	60% 30% 11,1% 0%
Selvi *et al*.,2012	Descompresión + Enucleación con curetaje (2) (9,1%) Enucleación con curetaje (20) (90,9%)	12--72	1 2	50% 10%
MacDonald *et al*., 2013	Enucleación (25) (86,2%) Enucleación + Crioterapia (3) (10,3%) Resección (1) (3,4%)	60	16 2 0	62% 70% 0%
Levorová *et al*., 2015	Enucleación + Curetaje (11) (50%) Enucleación + Curetaje + Solución de Carnoy (11) (50%)	36	5 5	45,4% 45,4%
Gupta *et al*., 2016	Enucleación (9) (30%) Enucleación + Soluión de Carnoy (3) (10%) Marsupialización (8) (26,7%) Resección (10) (33,3%)	12--60	5 1 3 0	55,5% 33,3% 37,5% 0%
Ledderhof *et al*.,2016	Enucleación + Osteotomía periférica + Solución de Carnoy (21) Enucleación + Osteotomía periférica + 5-fluorouracilo (11)	60	4 0	19% 0%
Leung YY *et al*., 2016	Enucleación + Solución de Carnoy (105) (100%)	10 -- 124	12	11.40%
Dias G *et al*., 2016	Marsupialización (10) Enucleación (69) Enucleación + Solución de Carnoy (2) Enucleación + Osteotomía periférica (11) Enucleación + Osteotomía periférica + Solución de Carnoy (22) Resección en bloque (3) Técnica quirúrgica dos tiempos (2)	64,8 64,2 63,6 63,6 52,8 63,6 42	4 18 1 2 0 0 0	40% 26,08% 50% 18,2% 0% 0% 0%
Santos de Castro *et al*., 2017	Marsupialización (99) (7,5%) Marsupializacón + Enucleación (45) (3,4%) Descompresión (27) (2%) Descompresión + Enucleación (101) (7,7%) Enucleación simple (1049) (79,4%)	60.1	18 8 5 12 218	18,2% 17,8% 18,5% 11,9% 20,8%
Nyimi Bushabu F *et al*., 2019	Mandibulectomía segmentaria (31) (88,57%) Mandibulectomía marginal (4) (11,43%)	60	0 1	0% 25%
Jung *et al*., 2021	Enucleación (203) Descompresión + Enucleación (67) Resección en bloque (4)	7 - 120	55 24 0	27,1% 35,8% 0%

## DISCUSIÓN

El queratoquiste odontogénico es una patología que debe tenerse en cuenta por su predominancia en la zona mandibular. Inicialmente, autores como Cavalieri *et al*. ^11^ manifiestan que estas lesiones pueden ser tratadas con biopsia incisional y descompresión, mediante un drenaje de polietileno que permite la reducción de la cavidad quística y facilita su posterior enucleación. Gracias a la revisión, podemos determinar que se trata de un tratamiento poco efectivo, ya que representa una recidiva del 17,8%.

Para la cirugía oral y maxilofacial, resulta de vital importancia reconocer los diferentes tipos de tratamientos quirúrgicos que se pueden emplear para tratar esta patología. Gracias a esta revisión de literatura, se obtuvo datos que indican la efectividad de cada uno de ellos, tal es el caso de autores como Selvi *et al*. ^15^, MacDonald *et al*. ^16^, Gupta *et al*. ^18^ y Jung *et al*. ^24^, quienes emplearon la resección en bloque y presentaron un 0% de recidiva. Sin embargo, otros tratamientos menos agresivos, como la enucleación más aplicación de solución de Carnoy, según Ribeiro *et al*. ^12^, mostraron tan solo un 4,55% de recidiva.

Entre los abordajes terapéuticos efectivos para el tratamiento del QQO, según Arenas de Frutos *et al*. ^9^, tenemos la enucleación simple, la enucleación más aplicación de la solución de Carnoy, la marsupialización y la resección como los más utilizados ante la presencia del queratoquiste. Estos tratamientos mostraron en nuestra revisión, según Dias *et al*. ^21^, un índice de recidiva del 26,08%, 50%, 40% y 0%, respectivamente, en relación con el número de pacientes tratados; esto nos ayuda a deducir que, si bien la enucleación simple sigue siendo el tratamiento más empleado, sigue presentando altos índices de recidiva.

Aunque varios métodos adyuvantes se han presentado para evitar la recidiva, la crioterapia y la aplicación de 5-fluorouracilo han sido las menos mencionadas y los únicos tratamientos coadyuvantes que no pudieron ser comparados con otro estudio ^16^^,^^19^.

## CONCLUSIONES

Gracias a la presente revisión, se pudo determinar que la resección en bloque es el tratamiento más efectivo ante la presencia de un QQO; sin embargo, se debe considerar que el uso de tratamientos conservadores más terapias adyuvantes de forma repetida en una lesión puede hacer menos traumático el manejo de esta patología, al presentar un menor deterioro estético y funcional del macizo facial.

### Caso clínico

Se presenta el caso de una paciente de sexo femenino que, en su tercera década de vida, acudió a consulta privada con antecedentes de queratoquiste odontogénico y manifestó molestias en la zona anterior de la mandíbula, específicamente en la sínfisis del mentón. Al realizar la exploración extraoral, los tejidos blandos presentaron una apariencia normal sin asimetrías; sin embargo, la exploración intraoral reveló un leve aumento de volumen, lo cual produjo la sospecha de una lesión quística.

**Figura 5:**
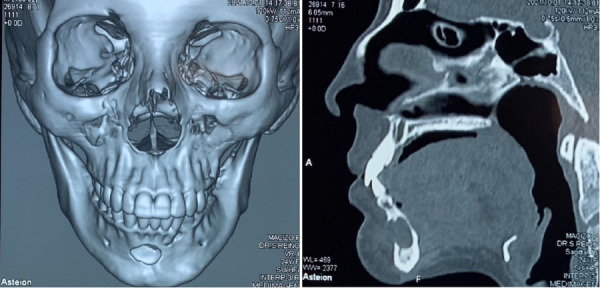
A y B) Estudios de imagen previos al tratamiento quirúrgico. A) Vista general de la TAC (tomografía axial computarizada). B) Corte sagital, visualización de zona hipodensa con erosión de tabla vestibular y adelgazamiento de tabla lingual.

La tomografía computarizada reveló una zona maxilar superior sin alteraciones, mientras que en la zona mandibular se pudo evidenciar una zona hipodensa de 3 x 2 x 3 cm que perforaba de manera cortical vestibular la región mentoniana (Fig. 5).

**Figura 6:**
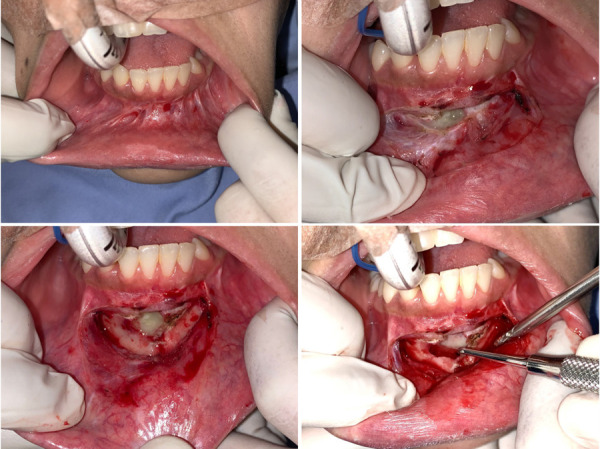
A y B) Abordaje quirúrgico mediante separación de la mucosa y aplicación de anestésico local (lidocaína al 2%); incisión con electrobisturí y exposición del queratoquiste. C y D) Identificación de la lesión.

Posteriormente, se realizó el abordaje quirúrgico de la lesión bajo anestesia general e infiltración de lidocaína al 2% en la zona a tratar. El acto quirúrgico consistió en realizar la enucleación de la lesión, el curetaje, la osteotomía periférica y la aplicación de injerto óseo, para finalizar con la aproximación de tejidos (Figs. 6 y 7). El quiste extraído fue enviado al servicio de anatomía patológica, donde se confirmó como una recidiva de queratoquiste odontogénico (Fig. 8).

**Figura 7:**
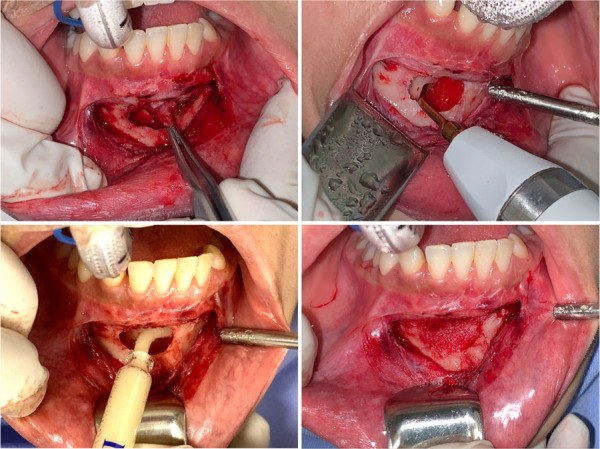
A) Enucleación del queratoquiste. B) Osteotomía periférica y regularización de la cavidad quística con ayuda de piezosurgery. C y D) Aplicación de aloinjerto óseo.

**Figura 8:**
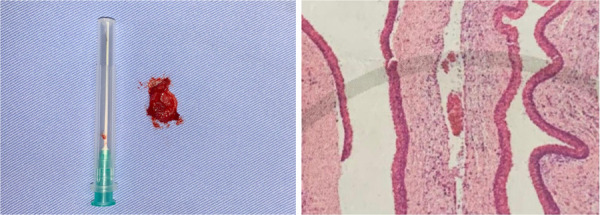
A) Material quístico comparado con una aguja de carga. B) Fotografía microscópica de la lesión, en la que se observa quiste de revestimiento epitelial uniforme con una capa de células basales hipercrómicas en empalizada compuesta por células cuboidales a columnares; a nivel del lumen hay presencia de restos queratináceos. El estroma adyacente presenta un leve proceso inflamatorio crónico moderado de predomio linfoplasmocitario, con estroma mixoide asociado a edema estromal.

La técnica para el abordaje quirúrgico se eligió debido a que, en una paciente joven, realizar una resección en bloque crearía una deformidad importante y la pérdida de órganos dentarios; por ello, se deberán realizar controles estrictos y exámenes radiográficos constantes para verificar lesiones recidivantes de forma oportuna y tratarlas en caso aparezcan.

## Fuente de financiamiento:

Esta investigación fue autofinanciad
